# Modeling and Characterization of Capacitive Coupling Intrabody Communication in an In-Vehicle Scenario

**DOI:** 10.3390/s19194305

**Published:** 2019-10-04

**Authors:** Yuan Xu, Zhonghua Huang, Shize Yang, Zhiqi Wang, Bing Yang, Yinlin Li

**Affiliations:** School of Mechatronical Engineering, Beijing Institute of Technology, Beijing 100081, China; 2120170269@bit.edu.cn (Y.X.); huangzh@bit.edu.cn (Z.H.); 2120170272@bit.edu.cn (S.Y.); wzq90@outlook.com (Z.W.); yangbinghyz@bit.edu.cn (B.Y.)

**Keywords:** capacitive coupling IBC, channel modeling, transmission path loss, finite element method (FEM), in-vehicle

## Abstract

Intrabody communication (IBC) has drawn extensive attention in the field of ubiquitous healthcare, entertainment, and more. Until now, most studies on the modeling and characterization of capacitive coupling IBC have been conducted in open space, while influences when using metallic-enclosed environments such as a car, airplane, or elevator have not yet been considered. In this paper, we aimed to systematically investigate the grounding effect of an enclosed metal wall of a vehicle on the transmission path loss, utilizing the finite element method (FEM) to model capacitive coupling IBC in an in-vehicle scenario. The results of a simulation and experimental validation indicated that the system gain in an in-vehicle scenario increased up to 7 dB compared to in open space. The modeling and characterization achieved in this paper of capacitive coupling IBC could facilitate an intrabody sensor design and an evaluation with great flexibility to meet the performance needs of an in-vehicle use scenario.

## 1. Introduction

With the rapid development of biomedical sensors and wearable devices in recent years, intrabody communication (IBC) has become an extensive research topic in the area of healthcare, entertainment, and so on [1,2,3].Unlike traditional wireless communication, which propagates through air channels, capacitive IBC uses human body tissue as the signal transmission channel, enabling potentially higher-bandwidth wearable applications with less power consumption, which plays a key role in small battery-powered wearable devices [4,5,6,7]. 

In capacitive coupling IBC, a transmitter (TX) transfers energy across the conductive human body by coupling energy to the body via a signal electrode (SE) and to the environment via a ground electrode (GE). The transmitted signal is then collected by a receiver SE [6,8]. In the coupling process, the transmitter (TX) and receiver (RX) ground electrodes along with the environment form a return path [9]. As the GEs remain floating, the path gain and transmission width are easily affected by the noisy environment because the signal return path is closed through the surroundings and external ground. Therefore, channel modeling is used to analyze the operation principle of capacitive human body communication (HBC) [10]. The transmission models are able to intuitively interpret the coupling mechanism of IBC and facilitate the transceiver design with great flexibility [10].

The modeling and characterization of capacitive coupling IBC has drawn much attention in recent years and can be categorized as a finite element method (FEM) model, a quasi-static field model, an equivalent electrical circuit model, or a distributed circuit model [11]. In Reference [12], an IBC channel model based on quasi-electrostatic field coupling was proposed, which described the transmission path loss generated at various distances and frequencies. In Reference [10], a system measurement of capacitively coupled IBC for a battery-powered transceiver was carried out, and a corresponding equivalent circuit model was proposed based on the electric field distribution around the human body. Mao et al. proposed a type of self-adapting capacitive coupling technique that compensates for the return path loss through a resonant matching method [13]. Ruoyu Xu et al. [14,15] used a finite element method (FEM) to simulate a capacitively coupled IBC channel model and studied the influence of the capacitive return path on the IBC channel and the interference of a nearby human body on IBC signal transmission. In Reference [16], the transmission characteristics of a capacitively coupled IBC in the frequency range of 100 kHz–100 MHz were measured using variously configured transceiver electrodes. In Reference [17], multipath propagation parameter estimation and modeling for channel measurement were performed. 

However, none of these studies about the modeling and measurements of capacitive coupling IBC were performed in a metallic-enclosed environment. A wearable device is supposed to be carried around and function reliably in use scenarios such as in-vehicle scenarios (e.g., a car, airplane, or elevator) [18]. In these environments, the electric field on the human body generated by the TX may be coupled to one or multiple sides of the metallic cabinet, instead of coupling only to the ground plane or an adjacent conductive object in an open space. As a result, the transmission characteristics of the return path become different from those of the open space use. However, there has been a lack of research about IBC channel characterization in the situation of the enclosed environment. It is unclear whether an IBC system designed under the assumption of an enclosed environment can work properly or how the transmission gain and width will be influenced in such environments. Therefore, this paper attempted to model and study the characterization of capacitive coupling IBC in an in-vehicle scenario. Through an analysis of the distribution of an electric field, an equivalent circuit model and a channel transfer function in accordance with the electric field coupling path in the electrode, human body, and cabinet walls are proposed. On the basis of the achieved channel transfer function, the variation in the channel attenuation was analyzed. The finite element method (FEM) was then applied to analyze the influence of channel parameters on the transmission gain with respect to different conditions, e.g., the height of the electrode, the distance between the electrode and the cabinet walls, etc. In addition, a pair of transmitter and receiver prototypes powered by battery were developed to validate the theoretical model and findings. 

The paper is organized as follows: Section 2 presents the theory of a capacitive coupling IBC system in an in-vehicle scenario. In Section 3, the FEM model of the capacitive coupling IBC in the vehicle is established and simulated, and the grounding effect of the cabinet walls is evaluated from the perspective of electric field distribution. Section 4 presents and discusses the experimental results achieved in a car using a battery-powered prototype transmitter. Section 5 draws the final conclusions of this paper.

## 2. Capacitive Coupling IBC System

**Figure 1:**
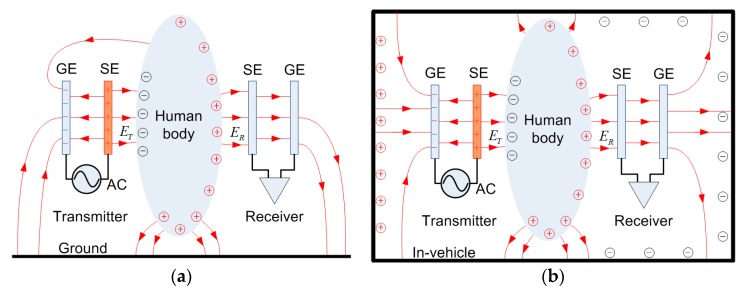
Illustration of capacitive coupling intrabody communication (IBC) system. (**a**) In open space, the electric fields are coupled to the ground as the return path. (**b**) In an in-vehicle scenario, the electric fields are coupled to multiple sides of the cabinet walls.

Conventional capacitive coupling IBC was put forward by T.G. Zimmerman in 1995 [19], as shown in Figure 1a. Two pairs of vertical electrodes were attached to the human body in order to transmit and receive signals. Typically, the signal electrodes (SEs) in the transmitter and receiver are attached to or in the vicinity of the human body, and the ground electrodes (GE) float. When the transmitter is applied with an alternating signal source, the coupling electric field *E_T_* between the SE and the body is established. On the receiver side, the SE detects the strength of the electric field *E_R_* and converts it into the displacement current, written as

(1) $$i_{r}=\frac{dQ}{dt}=\varepsilon_{0}\int_{S}\frac{dE_{r}}{dt}dS,$$

where *ε*_0_ is the permittivity, *S* is the area of the receiver SE, and *E* is the detected electric field strength. As a result, the transmission gain through the human body can also be expressed as follows [20]:

(2) $$G_{E}\left(\mathrm{dB}\right)=20\log_{10}\left(\frac{E_{r}}{E_{T}}\right).$$

A capacitive coupling IBC in a metallic-enclosed environment is shown in Figure 1b. Under the influence of an electric field generated by a transmitter, the cabin wall in the vicinity of the transmitter SE will be electrically induced and charged. In the open space scenario, the electric field line can return to the ground electrode, mostly through the ground. However, in in-vehicle conditions, the ground electrodes (GEs) and metal cabinet walls couple in various directions, which alters the returning path, which is indicated as the inductive electric field line surrounding the human body. Therefore, the coupling effect of the vehicle metal wall on the transmission performance of the capacitive coupling IBC becomes considerable. In order to further study the influence of the metal wall of the vehicle on the IBC in an in-vehicle scenario, we established an electric field coupling model to evaluate the transfer function of the system and thus provide a theory for a communications system and parameter optimization. 

### 2.1. Capacitive Coupling IBC Model in a Metallic-Enclosed Environment

**Figure 2:**
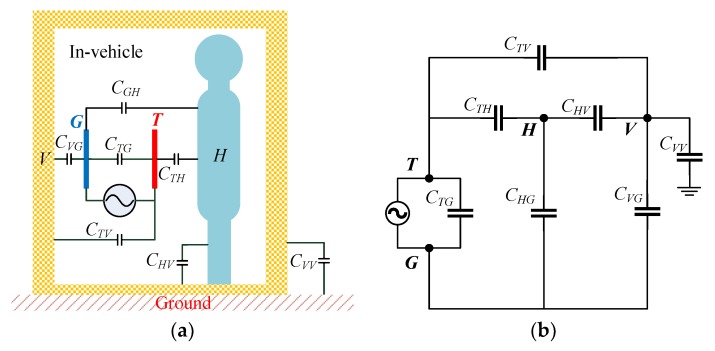
(**a**) Equivalent capacitance model of IBC system in a metallic-enclosed environment. (**b**) Equivalent circuit model of an IBC system.

The electric field induced by a human body is mainly determined by the capacitance among the transmitter, the human body, and the environment. In a metallic-enclosed cabinet, the coupling between an electrode and metallic walls can be modeled as a capacitance network, as shown in Figure 2a. The equivalent circuit model of the capacitance network is then constructed, as in Figure 2b.

Figure 2a shows a multiconductor model composed of a transmitter ground electrode, a signal electrode, a human body, and cabinet walls, wherein the potential and the electric charge of the ground electrode are respectively represented by *V_G_* and *Q_G_*; the potential and the electric charge of the signal electrode are denoted as *V_T_* and *Q_T_*, respectively; the potential and the electric charge of the human body are respectively *V_H_* and *Q_H_*; and the potential and the electric charge of the cabinet walls are respectively *V_V_* and *Q_V_.*

The relation between the electric charge and the potential of each conductor in the system can be expressed as follows:

(3) $$\{\begin{array}{c} Q_{G}=C_{GG}V_{G}+C_{GT}V_{T}+C_{GH}V_{H}+C_{GV}V_{V}\\ Q_{T}=C_{TG}V_{G}+C_{TT}V_{T}+C_{TH}V_{H}+C_{TV}V_{V}\\ Q_{H}=C_{HG}V_{G}+C_{HT}V_{T}+C_{HH}V_{H}+C_{HV}V_{V}\\ Q_{V}=C_{VG}V_{G}+C_{VT}V_{T}+C_{VH}V_{H}+C_{VV}V_{V}\\ Q_{G}=-Q_{T}\\ U_{in}=V_{T}-V_{G} \end{array},$$

where *C_ij_* is the mutual capacitance of conductor *i* upon conductor *j* and is defined as the charge carried by conductor *j* when other conductors are grounded and the potential of conductor *i* is increased to 1 V, where *C_ij_ = C_ji_*. *C_ii_* is the ground capacitance of conductor *i*, and *U_in_* is the excitation signal amplitude of the transmitter.

Conductor *T* and conductor *G* respectively correspond to the signal electrode and the ground electrode in the intrabody communication. The two conductors are near each other, placed on the surface of the human body. The signal source is connected between the signal electrode and the ground electrode. The coupling capacitance *C_GH_* and *C_TH_* between the two electrodes of the transmitter and the human body is neglectable. In the in-vehicle scenario, the cabinet walls are suspended above the ground, and the electric charge *Q_V_* is treated as zero. Due to electrostatic shielding by the enclosed metal space of the vehicle cabinet, the capacitance *C_ii_* of each conductor is regarded as zero.

In such a case, the above Equation (3) can be simplified as follows:

(4) $$\{\begin{array}{c} Q_{G}=C_{GV}V_{V}\\ -Q_{G}=C_{TH}V_{H}+C_{TV}V_{V}\\ \begin{array}{c} 0=C_{TH}V_{T}+C_{HV}V_{V}\\ 0=C_{GV}V_{G}+C_{TV}V_{T}+C_{HV}V_{H}+C_{VV}V_{V} \end{array} \end{array}.$$

The above formula is solved to obtain

(5) $$V_{H}-V_{V}=\frac{C_{GV}\left(C_{GV}+C_{TV}+C_{TH}\right)}{2\left(C_{GV}C_{HV}+C_{TV}C_{HV}\right)-C_{TH}C_{VV}}U_{in}.$$

### 2.2. Detection System Model

**Figure 3:**
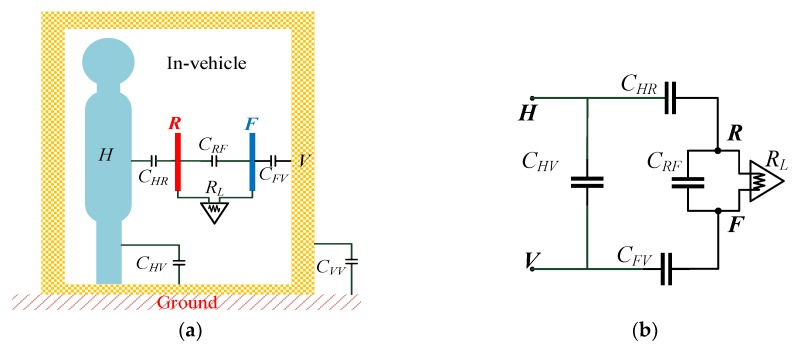
(**a**) Diagram of an equivalent capacitance model of the receiver. (**b**) Equivalent circuit model of the receiver.

The aforementioned result shows that the enclosed space of the cabinet walls can affect the electric coupling strength in the transmitter, the human body, and the environment and accordingly further influence the human–body electric field distribution. To quantitatively evaluate the influence, we established a receiver circuit model to analyze the path gain of the capacitive coupling IBC in an in-vehicle scenario. The receiver model is shown in Figure 3a.

When the receiver is used in a metallic-enclosed environment, the signal electrode *R* detects the change of the electric field near the human body and generates an induced current through the receiver circuit. An equivalent circuit model is shown in Figure 3b, and the output signal is as follows:

(6) $$U_{O}=\frac{Z_{RF}}{Z_{HR}+Z_{RF}+Z_{FV}}\left(V_{H}-V_{V}\right).$$

The transfer function of the system is then obtained as

(7) $$G\left(\mathrm{dB}\right)=20\log_{10}\left(\frac{U_{o}}{U_{in}}\right)=20\log_{10}\left(\frac{Z_{RF}}{Z_{HR}+Z_{RF}+Z_{FV}}\cdot \frac{C_{GV}\left(C_{GV}+C_{TV}+C_{TH}\right)}{2\left(C_{GV}C_{HV}+C_{TV}C_{HV}\right)-C_{TH}C_{VV}}\right).$$

## 3. Simulation of Capacitive Coupling IBC in an In-Vehicle Scenario

In order to more realistically simulate and analyze the shielding effect of the vehicle on the transmission gain, the finite element simulation method was adopted to obtain the capacitance parameters of the typical positions. Subsequently, the results obtained were put into the above transfer function to evaluate the amplitude characteristics. The simulation results were then compared to the theoretical model results under the same conditions.

ANSYS Maxwell was used to model the capacitive coupling IBC in a vehicle. Maxwell is an FEM simulation software that performs electromagnetic simulations in a limited area with a defined boundary condition. In this section, we will detail how to establish an FEM model and quantitatively compare the differences in electric field distributions around the human body in open space and in an in-vehicle scenario.

### 3.1. FEM Simulation Model and Setup

**Figure 4:**
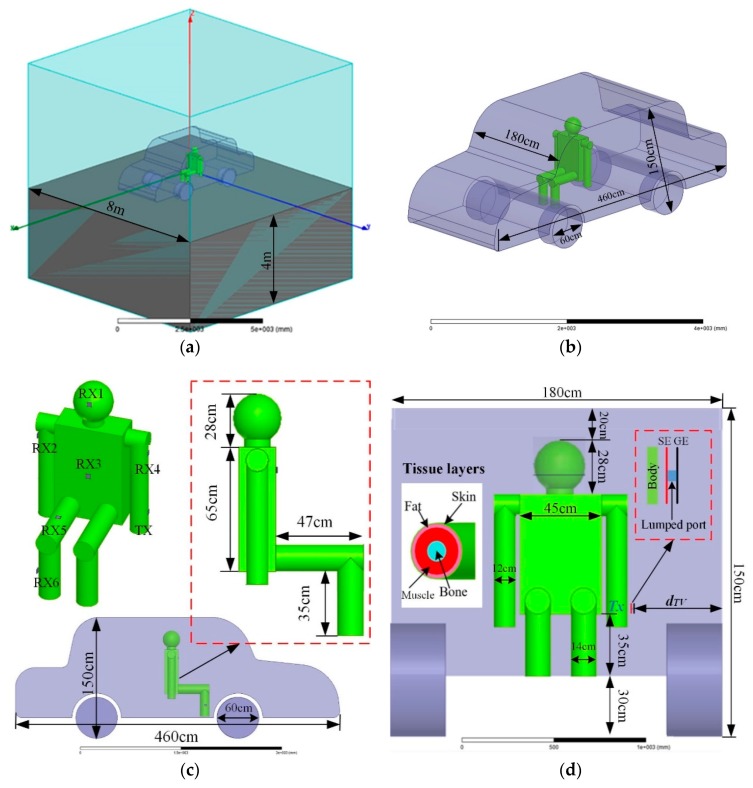
Finite element method (FEM) model and simulation environment. (**a**) Simulation environment of the IBC in the vehicle. (**b**) A simulation of the vehicle model size. (**c**) Left view of the human body in the in-vehicle scenario. (**d**) Front view of the human body in the in-vehicle scenario.

The entire FEM simulation environment is shown in Figure 4, which includes the external ground plane; the radiation boundary in the air, vehicle, and human body; and the transmitter electrodes. The ground was modeled as a box, and the top surface was set as an infinite ground plane with a size of 8 × 8 × 4 m^3^. The outer boundary of the calculation region was set to radiation. 

A simulation model of a human body in a vehicle is illustrated in Figure 4b. The car model had a metal cavity with a thickness of 3 cm, which refers to a typical car size. The human body remained in a sitting position in the vehicle, and it was composed of a head, a neck, a torso, arms, and legs. To ensure simulation results are close to a realistic situation, the dielectric properties of different human tissue layers should be considered. The human body has a complicated internal organizational structure: different physiological tissues may have a different conductivity (*σ*) or a different relative permittivity (*ε_r_*), and such factors change with frequency [21,22]. Table 1 shows the conductivity and the relative permittivity of human body tissues under several typical frequencies [21]. Table 2 summarizes the thickness of the tissue layers in different body parts [15]. The torso was 35 cm high from the chassis, and other dimensions are shown in Figure 4c,d.

The two electrodes of the transmitting terminal were arranged parallel to the human body and cabinet walls, so *C_TH_*, *C_TG_*, and *C_GV_* were approximately equivalent to the parallel plate capacitor [9] (according to the IBC equivalent capacitor model in the in-vehicle scenario in Figure 2). In order to increase system gain, it was necessary to maximize the coupling area between the two electrodes and the human body or cabinet walls. Moreover, the influence of differently shaped electrodes on human body transmission characteristics was simulated in Reference [13]. The result showed that a square electrode had a better effect than did an equilateral triangle electrode or a round electrode due to the fringe effect of the parallel plate capacitor [23]. In practical applications, a transmitter is worn on such body parts as a wrist or a calf. The coupling area between a signal electrode and a human body is about 20–30 cm^2^, so a 5 × 5 cm^2^ square electrode was selected for this paper. 

The influence of a standard self-adhesive Ag/AgCl electrode with conductive paste and a copper electrode on human body transmission characteristics was tested in Reference [16]. The result showed that a self-adhesive electrode smeared with conductive paste had better transmission characteristics. However, the conductive slurry adopted on the self-adhesive electrode could come in direct contact with the skin and accordingly could lead to skin allergy patients [24]. Furthermore, the influence resulting from electrode input impedance caused by a metal electrode on the skin could be ignored. This indicated that even if conductive paste was not smeared to resist contact resistance, the metal electrode could also stably communicate with the human body [25,26]. Therefore, a copper electrode, which has better adaptivity and is regarded as an approximately perfect conductor, was selected for this paper.

In consideration of the average maximum power of 0.08 W/kg borne by human body tissue [27], the transmitting power was set as 0 dBm and had a constant power output in order to ensure personal safety. The SE and GE were connected by a 50-Ω lumped port. Since 0 dBm corresponded with the transmitter output voltage of 0.224 V, we set the distance between SE and GE to 0.01 m, and the transmitted electric field strength was 22.4 V/m (27 dBV/m). In the simulation, as shown in Figure 4c, the transmitter was located in the left forearm (TX) and was placed parallel to the cabinet walls. According to the actual application scenario, the receiver position selected the head (RX1), the right upper arm (RX2), the chest (RX3), the left upper arm (RX4), the right thigh (RX5), and the right calf (RX6), which are six typical positions. For final product use, the copper electrode will be coated. In order to emulate the effect of the coating layer isolation distance on the path transmission gain, all of the signal electrodes of the transmitter and receiver were separated from the skin by a 0.2-cm-thick RF4 dielectric layer. 

In order to investigate the influence of the vehicle, the electric field detected in the receiver side was calculated with different *d_TV_* (distance between the transmitter and vehicle) and different receiver positions (RX1, RX2, RX3, RX4, RX5, and RX6). The simulation frequency sweeps ranged from 1 MHz to 50 MHz. 

### 3.2. Simulation Results and Discussion

**Figure 5:**
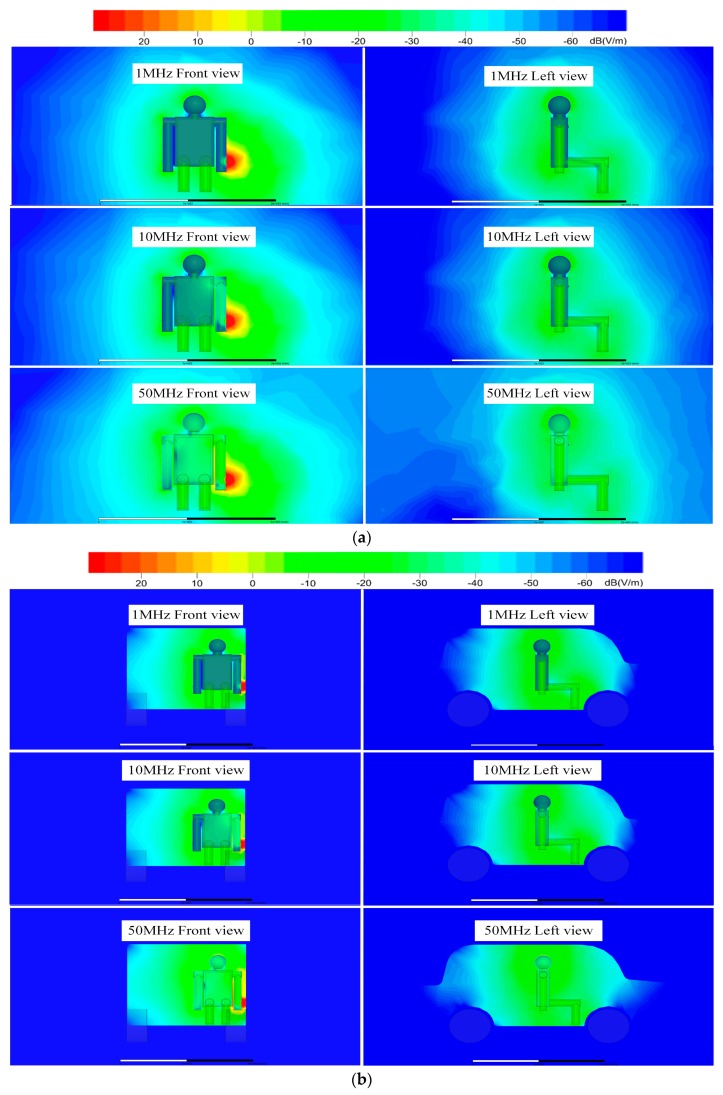

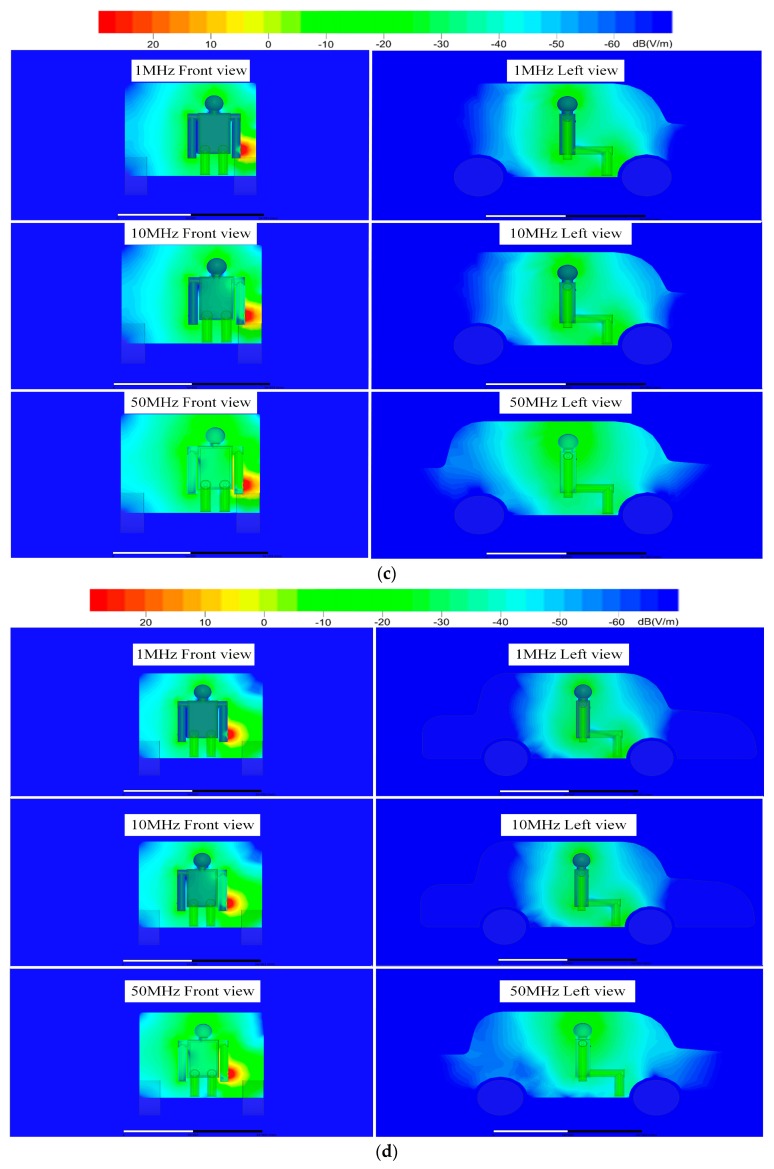
Electric field strength distribution around the human body at frequencies of 1, 10, and 50 MHz. (**a**) In open space; (**b**) *d_TV_* = 5 cm; (**c**) *d_TV_* = 20 cm; (**d**) *d_TV_* = 50 cm.

Figure 5 shows representative simulation results of the electric field strength distribution around the human body at frequencies of 1, 10, and 50 MHz. Figure 5a shows a front view and a left view of the field strength distribution in open space, and Figure 5b–d exhibits the electric field from the in-vehicle scenario with 5, 20, and 50 cm separation from the cabinet walls. When comparing the four different scenarios, it was obvious that the electric field shown in Figure 5b–d was stronger than the one in Figure 5a. Additionally, when the separation distance between the transmitter and the vehicle wall decreased, the electric field tended to be enhanced.

**Figure 6:**
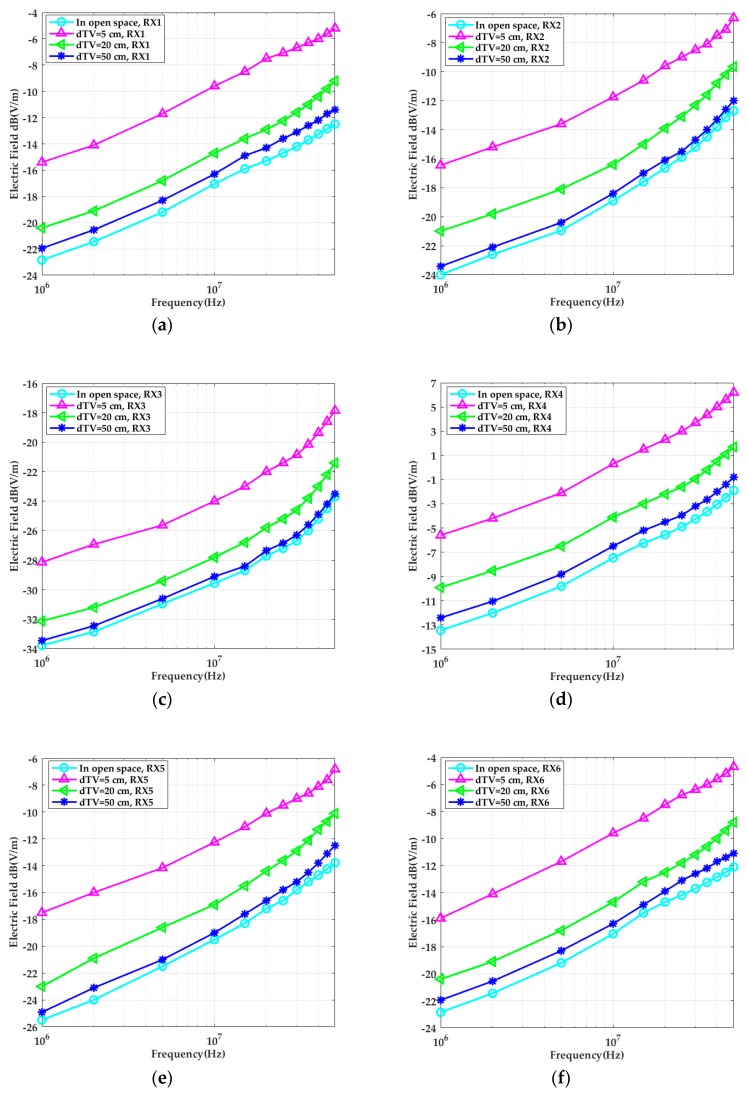
The simulated electric field strength when the transmitter was placed on the left forearm with different separation distances *d_TV_* from the vehicle. The electric field strengths at position (**a**) RX1 (head), (**b**) RX2 (right upper arm), (**c**) RX3 (chest), (**d**) RX4 (left upper arm), (**e**) RX5 (thigh), and (**f**) RX6 (calf) are presented.

As can be seen from Figure 6, when the distance between the transmitter and the cabinet walls *d_TV_* equaled 5 cm, compared to open space, the electric field strength received by each position of the human body was significantly enhanced by approximately 7 dB. As the distance further increased, the electric field strength continued attenuating. When *d_TV_* = 50 cm, the head (RX1) and the calf (RX6) were relatively closer to the upper and lower metal cabinet walls, and the electric field strength was slightly enhanced compared to in open space. However, the right upper arm (RX2) and the trunk (RX3) were relatively far from the surrounding cabinet walls, and their electric field strengths were less affected, which was similar to in open space.

**Figure 7:**
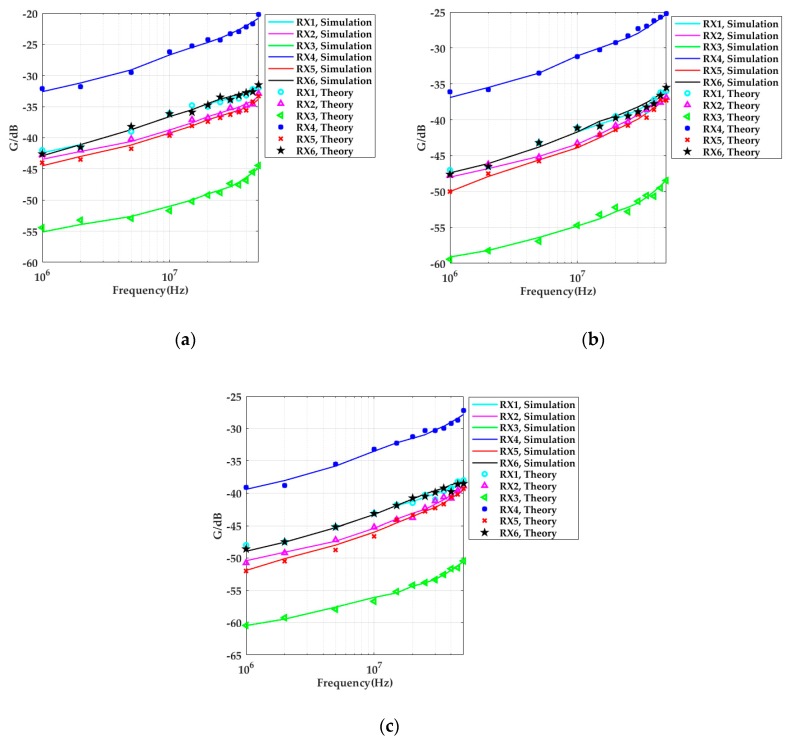
Comparison of system path gain in the simulation and theoretical models in the in-vehicle scenario: (**a**) *d_TV_* = 5 cm; (**b**) *d_TV_* = 20 cm; (**c**) *d_TV_* = 50 cm.

The capacitance parameters between the human body, the vehicle, and the transceiver were then obtained by finite element simulation in the ANSYS Maxwell software. The capacitance values were substituted into the system transfer function (Equation (7)), and the amplitude–frequency characteristics of the theoretical model were computed and compared to the simulation results. The theoretical values of the system gain in the in-vehicle scenario are shown in Figure 7. The results in Figure 6 were substituted into Equation (2), and the simulation results of the system gain at different frequencies were obtained, and we compared them to the theoretical values. The results demonstrate that the simulation was in accordance with the theoretical model.

**Figure 8:**
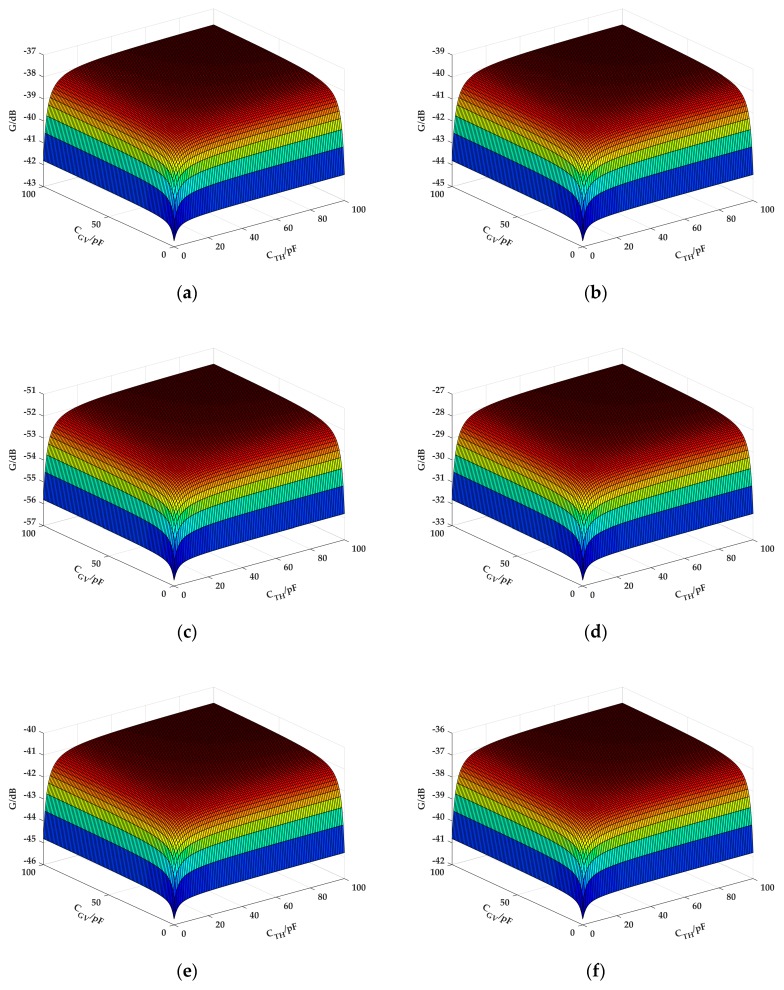
Influence of the coupling capacitances *C_TH_* and *C_GV_* on system path gain: (**a**) RX1 (head); (**b**) RX2 (right upper arm); (**c**) RX3 (chest); (**d**) RX4 (left upper arm); (**e**) RX5 (thigh); (**f**) RX6 (calf).

Meanwhile, the influences of the coupling capacitance *C_TH_* of the forward human body electric field and the coupling capacitance *C_GV_* of the signal circuit on the gain of the system were also studied. On the basis of the above simulation-achieved capacitance parameters, the frequency was set at 10 MHz, and *C_TH_* and *C_GV_* were set as the variables in the range 1–100 pF to emulate the system path gain of the six different receiving locations in the in-vehicle scenario. Their influences on the generation of the system gain are shown in Figure 8.

According to Figure 8, the coupling capacitances *C_TH_* and *C_GV_* had the same influence on system gain. Namely, when the capacitance was greater, the system gain was stronger. In addition, the influences of *C_TH_* and *C_GV_* on gain were associated with each other. If one coupling capacitance was very small, then the change in the other capacitance slightly influenced gain. This phenomenon was due to *C_TH_* and *C_GV_* being serially connected in the signal circuit, and the equivalent capacitance was as follows:

(8) $$\frac{C_{TH}C_{GV}}{C_{TH}+C_{GV}}\approx \frac{\varepsilon S}{d_{TH}+d_{GV}},$$

where *S* is the equivalent capacitive coupling area, *d_TH_* is the distance between the signal electrode (SE) and the human body, and *d_GV_* is the distance between the ground electrode (GE) and the cabinet walls. The results of Figure 8 further verified that when the signal electrode was placed close to the human body and the ground electrode was placed close to the cabinet walls, the received signal was strongest. When the distance between the transmitter and the vehicle cabinet walls or the human body increased, the channel gain was significantly attenuated.

## 4. Experimental Validation 

In order to further validate the coupling effect of the cabinet walls, comparison experiments using the prototype IBC system were conducted in open space and in in-vehicle scenarios.

### 4.1. Transceiver

**Figure 9:**
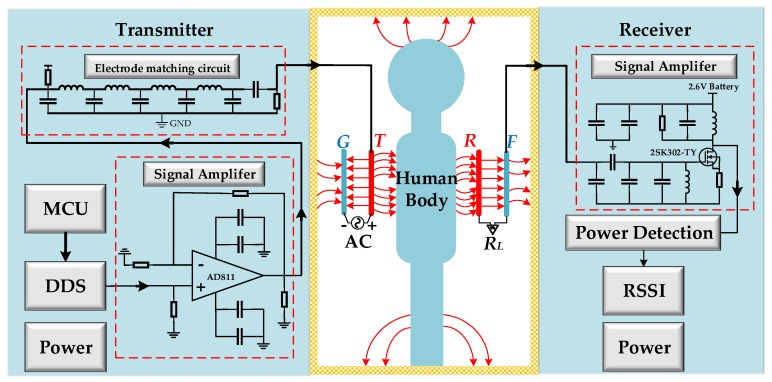
The functional block diagram of the experimental IBC system.

In an in-vehicle IBC scenario, data communication is realized through electric field coupling. In order to simulate realistic intrabody communication, the common ground of a transmitter and a receiver should be removed. For this purpose, batteries are used as the power supply for both the transmitter and the receiver. The experimental system diagram is shown in Figure 9.

In order to match the impedance between the electrode and the amplifier, an electrode-matching circuit was designed to obtain an approximately equal transmission gain up to a frequency of 200 MHz. For the signal amplifier circuit, a wideband operational amplifier (AD811) was chosen. The chip had a −3 dB bandwidth of 100 MHz, which was suitable for the broadband amplification requirements of this system. The receiver adopted the signal-amplifying circuit based on N-type field effect tubes (2SK302-TY), which had a low noise figure (*NF* = 1.7 dB), low reverse transfer capacitance (*C_rss_* = 0.035 pF), and a large common-source high-frequency power gain (*G_ps_* = 28 dB).

**Figure 10:**
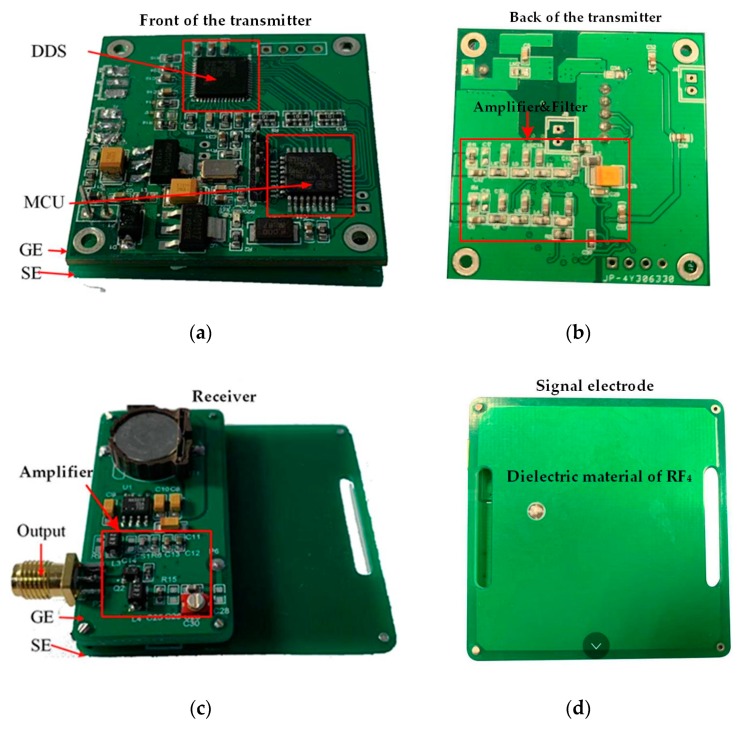
The prototype of the transceiver used in the experiment: (**a**) front of the transmitter; (**b**) back of the transmitter; (**c**) receiver; (**d**) signal electrode.

The transmitter, powered by a button battery, is shown in Figure 10a,b. A Tx device is a portable signal generator (SG) that was developed on the basis of an AD9959 generating sine wave through a direct digital frequency synthesizer (DDS). The device incorporates a high-speed 10-bit digital-to-analog converter (DAC) with an excellent wideband and a narrow-band spurious free dynamic range (SFDR). The DDS has a dedicated 32-bit frequency tuning word, 14 bits of phase offset, and a 10-bit output scale multiplier. The configuration codes are sent to a phase accumulator in the DDS by a microprogrammed control unit (MCU) (Stm32F1030), and the phase accumulator subsequently changes the phase value in the phase register in each clock period. By searching the sine wave table, a wave memorizer can convert phase information into amplitude information, and finally the sine wave is generated by the DAC. The DAC output voltage range is + 0.5 V to −0.5 V. The sine wave signal is connected to a signal electrode and subsequently coupled to the human body after being amplified. The SE and GE are connected by a 50-Ω lumped port. The programmable frequency provided by the DDS is in the range of 1–50 MHz, stepped by 1 KHz.

In the capacitive coupling IBC system, there are three equivalent impedances associated with the electrodes: the signal electrode (SE)–ground electrode (GE), the signal electrode (SE)–human tissues, and the impedance of each electrode itself [9]. Due to the dielectric properties of the human body, frequency has a great influence on the impedance of human tissues. In the experiment, we used the method of an analog test to realize the output power of the transmitter (0 dBm). We used a signal generator (Agilent 81150A) to output the sine wave signal of 0 dBm at a constant power: the signal electrode shown in Figure 10d was coupled to the human body, and the corresponding received power P1 was measured by the receiver. Then the transmitter (under the same conditions) was used for the same test, and the received power P2 was obtained. The amplitude of the output signal was adjusted by the DDS. When P1 = P2, the transmitter and the signal generator had the same output power. The other conditions of the two test methods were consistent. The signal generator and the transmitter had the same output frequency, and the receiver was fixed. At the same time, in order to ensure the grounding isolation of the system, an uninterruptible power system (UPS, APC RS1000) was used to power the signal generator. In the experiment, six receiving positions of three subjects were tested at different frequencies to ensure that the transmitter output signal was 0 dBm. The ground electrode (GE) size of the transmitter is 5 cm × 5 cm.

A vertically configured receiver electrode, shown in Figure 10c, was used to detect the electric field signal emitted from the source. The ground electrode (GE) size of the receiver is 2.5 cm × 5 cm. As shown in Figure 10d, the signal electrode (SE) of the transceiver consists of a pair of copper electrodes measuring 5 cm × 5 cm. The outside of the copper electrode was covered by a 0.2-cm-thick dielectric material of RF4, which was used to emulate the coating layer on the electrode of the final product. The interval between the SE of the transceiver and the GE is 1 cm.

### 4.2. Measurement Setup

**Figure 11:**
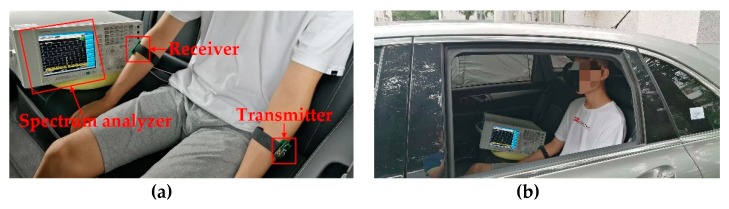
An illustration of the experimental setup in the in-vehicle scenario. (**a**) the local scenario; (**b**) the whole scenario.

**Figure 12:**
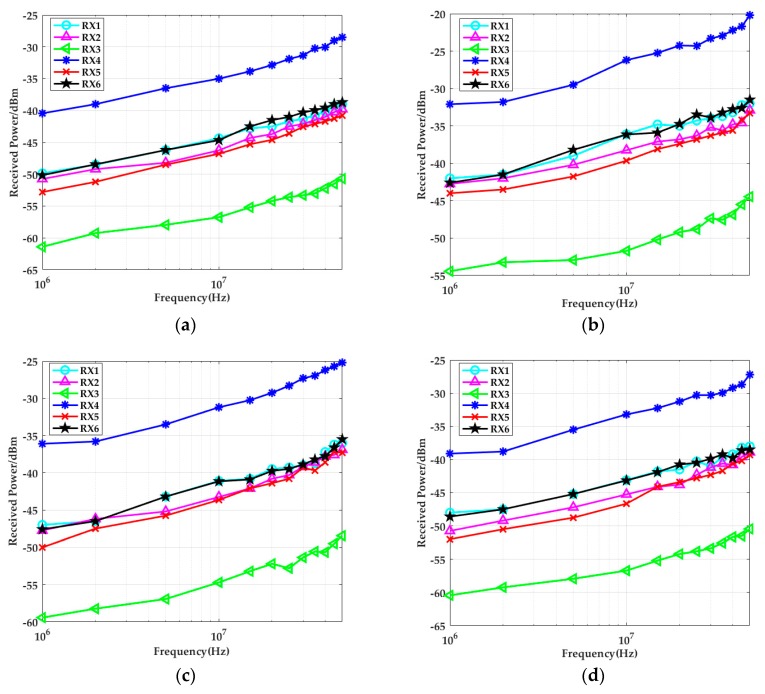
Comparison of the average received power of six typical positions of three subjects at different frequencies: (**a**) in open space; (**b**) *d_TV_* = 5 cm; (**c**) *d_TV_* = 20 cm; (**d**) *d_TV_* = 50 cm.

The measurements were conducted in in-vehicle and open space situations. The measurement setup in the in-vehicle scenario is shown in Figure 11. The experimental equipment included a battery-powered transceiver and a spectrum analyzer (Agilent N9030A), which were used to measure the received power. In addition, an uninterruptible power system (UPS, APC RS1000) was used to power the spectrum analyzer. Specifically, three male subjects (with heights of 171 cm, 174 cm, and 180 cm and body weights of 67.5 kg, 76 kg, and 81.5 kg) were selected for the experiment. As shown in Figure 12, the average values of the received power were measured in the three subjects. The transmitter was placed parallel to the left forearm of the human body, and the receivers were placed parallel to the head (RX1), the right upper arm (RX2), the chest (RX3), the left upper arm (RX4), the right thigh (RX5), and the right calf (RX6): they measured the received power at different distances between the transmitter and the car at the in-vehicle scenario.

### 4.3. Results and Discussion

The measured results of the two different scenarios are shown in Figure 12. Obviously, compared to open space, the received power was stronger when the system was in the in-vehicle scenario. On the one hand, when the distance between the transmitter and the cabinet walls was 5 cm, the received power of each body part was 7 dBm higher than in open space. With an increase in the distance between the transmitter and the cabinet walls, system gain attenuated. On the other hand, when the distance between the transmitter and the cabinet walls reached a certain value, the receiver (RX4) and the transmitter were on one side of the body, and they were close to each other, the received power was the strongest. Because the electric field strength was mainly concentrated on one side of the transmitter at that moment (where the body was influenced by inductive charge), the energy gathered on the nearby metallic cabinet walls. When the receiver got closer to the cabinet walls, the system gain became stronger. The measurement results in Figure 12d also proved this conclusion. When the body was relatively far from the cabinet walls, compared to the received power in open space, the received power in the head (RX1) and calf (RX6) (they were closer to the upper and lower cabinet walls) was a little stronger. The right upper arm (RX2) and the chest (RX3) were relatively far from the cabinet walls, so the received power was influenced less, which was almost equal to the measurement results in open space. 

When the simulation results shown in Figure 6 and the experimental results in Figure 12 were compared, both studies showed that the received signal strengths under the conditions of open space were significantly less than the values in the in-vehicle scenarios. In addition, it was found that the more the distance between the transmitter and the cabinet metallic wall increased, the smaller the received signal strength became. When the distance between the transmitter and the cabinet walls was 5 cm, the system gain was approximately 7 dB higher than in open space. When the body was relatively far from the cabinet walls, the system gains measured at the head (RX1) and calf (RX6) increased approximately 3 dB compared to in open space. The right upper arm (RX2) and the chest (RX3) were relatively far from the cabinet walls, and the system gain of the situation was less influenced, which was almost equal to the measurement results in open space. As it was influenced by the test environment, the overall system error of the simulation and experimental results was within 0.5 dB. When the input signal frequency was higher than 10 MHz, the difference between the two results sometimes reached 1 dB, which may have been due to parasitic effects.

## 5. Conclusions

In this paper, the transmission characteristics of a capacitive coupling IBC channel in an in-vehicle scenario were investigated. A theoretical model of capacitive coupling IBC in an in-vehicle scenario was established. An FEM simulation model in ANSYS Maxwell software was built to study the influence of a vehicle on the transmission electric field strength. In order to further validate the results, an experiment using an IBC prototype system was conducted to measure the receiving signal strength at six representative positions on the human body in the frequency range of 1–50 MHz. The experimental results were comparatively performed in open space and in an in-vehicle condition. The study showed that when the human body was close to the vehicle cabinet walls, the system gain increased by approximately 7 dB. 

In conclusion, the system path gain increased when the IBC system worked in the in-vehicle scenario, especially when the transceiver was in the vicinity of the metallic wall and tightly coupled to the cabinet. The modeling and characterization of the capacitive coupling IBC achieved in this paper can facilitate an intrabody sensor design and an evaluation with great flexibility to meet the performance needs of an in-vehicle use scenario.

## Figures and Tables

**Table 1:** Relative permittivity and conductivity of human tissues at typical frequencies.

Tissues	Relative Permittivity *ε_r_*					Conductivity *σ* (S/m)				
	1 MHz	5 MHz	10 MHz	20 MHz	50 MHz	1 MHz	5 MHz	10 MHz	20 MHz	50 MHz
skin	823	427	362	168	114	0.01	0.20	0.21	0.25	0.04
fat	36	17	14	12	11	0.03	0.03	0.03	0.03	0.04
muscle	1850	363	171	95	32	0.51	0.6	0.62	0.64	0.68
bone	187	159	112	109	107	0.01	0.01	0.02	0.02	0.03

**Table 2:** Thicknesses of tissue layers (mm).

Tissues	Head	Neck	Torso	Arm	Leg
skin	1.26	1.26	1.26	1.26	1.26
fat	2	8.74	8.74	8.74	8.74
muscle	2	42	30	28	34
bone	10	23	20	22	26
